# *N*,*N*′-Bis(6-methyl-2-pyrid­yl)oxamide

**DOI:** 10.1107/S1600536809040902

**Published:** 2009-10-17

**Authors:** Pei-Chi Cheng, Chia-Jun Wu, Jhy-Der Chen

**Affiliations:** a Department of Chemistry, Chung-Yuan Christian University, Chung-Li, Taiwan

## Abstract

In the crystal structure of the title compound, C_14_H_14_N_4_O_2_, the mol­ecules are almost planar (mean deviation 0.028 Å) and a weak intra­molecular N—H⋯O hydrogen bond between the H atom bound to an oxamide N atom and a carbonyl O atom is found. The asymmetric unit consits of one half-mol­ecule which is located on a centre of inversion.

## Related literature

For the synthesis, see: Siedel *et al.* (1970 ▶). For a series of Ag(I) coordination polymers containing *N^1^*,*N^2^*-bis­(2-pyrid­yl)­oxamide ligands, see: Hsu & Chen (2004 ▶); Hu *et al.* (2004 ▶).
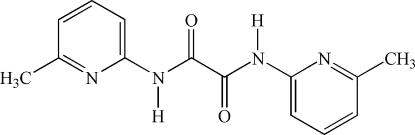


## Experimental

### 

#### Crystal data

C_14_H_14_N_4_O_2_
*M* *_r_* = 270.29
Monoclinic, 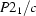

*a* = 3.8925 (6) Å
*b* = 15.964 (2) Å
*c* = 10.8353 (14) Å
β = 94.461 (13)°
*V* = 671.26 (16) Å^3^
*Z* = 2
Mo *K*α radiation
μ = 0.09 mm^−1^
*T* = 295 K
0.4 × 0.2 × 0.1 mm

#### Data collection

Bruker P4 diffractometer
Absorption correction: multi-scan (*XSCANS*; Siemens, 1995 ▶) *T*_min_ = 0.741, *T*_max_ = 0.762
1867 measured reflections
1190 independent reflections
767 reflections with *I* > 2σ(*I*)
*R*_int_ = 0.028
3 standard reflections every 97 reflections intensity decay: none

#### Refinement

*R*[*F* ^2^ > 2σ(*F* ^2^)] = 0.040
*wR*(*F* ^2^) = 0.101
*S* = 1.01
1190 reflections
93 parameters
H-atom parameters constrained
Δρ_max_ = 0.14 e Å^−3^
Δρ_min_ = −0.12 e Å^−3^

### 

Data collection: *XSCANS* (Siemens, 1995 ▶); cell refinement: *XSCANS*; data reduction: *SHELXTL* (Sheldrick, 2008 ▶); program(s) used to solve structure: *SHELXS97* (Sheldrick, 2008 ▶); program(s) used to refine structure: *SHELXL97* (Sheldrick, 2008 ▶); molecular graphics: *SHELXTL*; software used to prepare material for publication: *SHELXTL*.

## Supplementary Material

Crystal structure: contains datablocks I, global. DOI: 10.1107/S1600536809040902/nc2161sup1.cif

Structure factors: contains datablocks I. DOI: 10.1107/S1600536809040902/nc2161Isup2.hkl

Additional supplementary materials:  crystallographic information; 3D view; checkCIF report

## Figures and Tables

**Table 1:** Hydrogen-bond geometry (Å, °)

*D*—H⋯*A*	*D*—H	H⋯*A*	*D*⋯*A*	*D*—H⋯*A*
N2—H2*A*⋯O^i^	0.86	2.24	2.6718 (18)	111

Symmetry code: (i) 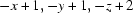.

**Fig. 1.**
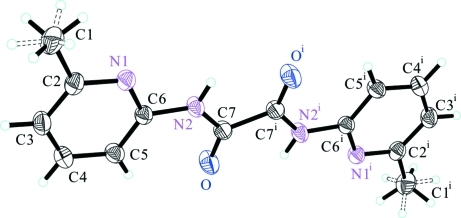
Crystal structure of the title compound with labeling and displacement ellipsoids drawn at the 30% probability level. Symmetry code: i = -x + 1, -y + 1, -z + 2. The disorder is shown with open bonds.

## supplementary crystallographic information

### Comment

A series of Ag(I) coordination polymers containg *N^1^*,*N^2^*-bis(2-pyridyl)oxamide ligands have been prepared, which show one-dimensional and two-dimensional structures (Hsu, *et al.*, 2004; Hu, *et al.*, 2004). To investigate the steric effect of the alkyl groups on the structural type of such coordination polymers, we have synthesized the title compound. Within this project its crystal structure was determined.

In its crystal structure weak intramolecular N-H···O hydrogen bonding is found (Tab. 1) and the molecules are almost planar (Fig. 1).

### Experimental

2-Amino-6-methylpyridine (6.2 g, 57.3 mmol) was dissolved in 200 ml CH_2_Cl_2_, followed by addition of triethyl amine (10.0 ml, 72.1 mmol) at 0° C. The mixture was then stirred for 10 min. Oxalyl chloride (2.5 ml, 28.7 mmol) in 10 ml CH_2_Cl_2_ was then added slowly to the above mixture. After continuous stirring for 3 h at 0° give maximu[C, 200 ml hexanes was added to the mixture to induce precipitate. The solid was filtered, washed with water to give a white product. Yield: 2.8 g (36%). Coloress plate crystals suitable for X-ray crystallography were obtained by slow evaporization of the solvent from a solution in CH_2_Cl_2_.

### Refinement

All the hydrogen atoms were placed into idealized positions and constrained by the riding atom approximation with *C*—H = 0.93 — 0.96 Å, N—H = 0.86 Å and *U*_iso_(H) = 1.5 *U*_eq_(C) or 1.2 *U*_eq_(C, N). The methyl H atoms are disordered and were refined in two different orientations.

### Crystal data

**Table:**

C_14_H_14_N_4_O_2_	*F*(000) = 284
*M**_r_* = 270.29	*D*_x_ = 1.337 Mg m^−^^3^
Monoclinic, *P*2_1_/*c*	Mo *K*α radiation, λ = 0.71073 Å
Hall symbol: -P 2ybc	Cell parameters from 23 reflections
*a* = 3.8925 (6) Å	θ = 7.5–12.6°
*b* = 15.964 (2) Å	µ = 0.09 mm^−^^1^
*c* = 10.8353 (14) Å	*T* = 295 K
β = 94.461 (13)°	Plate, colorless
*V* = 671.26 (16) Å^3^	0.4 × 0.2 × 0.1 mm
*Z* = 2	

### Data collection

**Table:**

Bruker P4 diffractometer	767 reflections with *I* > 2σ(*I*)
Radiation source: fine-focus sealed tube	*R*_int_ = 0.028
graphite	θ_max_ = 25.0°, θ_min_ = 2.3°
ω scans	*h* = −1→4
Absorption correction: multi-scan (*XSCANS*; Siemens, 1995)	*k* = −1→18
*T*_min_ = 0.741, *T*_max_ = 0.762	*l* = −12→12
1867 measured reflections	3 standard reflections every 97 reflections
1190 independent reflections	intensity decay: none

### Refinement

**Table:**

Refinement on *F*^2^	Secondary atom site location: difference Fourier map
Least-squares matrix: full	Hydrogen site location: inferred from neighbouring sites
*R*[*F*^2^ > 2σ(*F*^2^)] = 0.040	H-atom parameters constrained
*wR*(*F*^2^) = 0.101	*w* = 1/[σ^2^(*F*_o_^2^) + (0.0467*P*)^2^] where *P* = (*F*_o_^2^ + 2*F*_c_^2^)/3
*S* = 1.01	(Δ/σ)_max_ < 0.001
1190 reflections	Δρ_max_ = 0.14 e Å^−^^3^
93 parameters	Δρ_min_ = −0.11 e Å^−^^3^
0 restraints	Extinction correction: *SHELXL97* (Sheldrick, 2008), Fc^*^=kFc[1+0.001xFc^2^λ^3^/sin(2θ)]^-1/4^
Primary atom site location: structure-invariant direct methods	Extinction coefficient: 0.021 (4)

### Special details

**Table:**

Experimental. Refinement of *F*^2^ against ALL reflections. The weighted *R*-factor *wR* and goodness of fit *S* are based on *F*^2^, conventional *R*-factors *R* are based on *F*, with *F* set to zero for negative *F*^2^. The threshold expression of *F*^2^ > σ(*F*^2^) is used only for calculating *R*-factors(gt) *etc*. and is not relevant to the choice of reflections for refinement. *R*-factors based on *F*^2^ are statistically about twice as large as those based on *F*, and *R*- factors based on ALL data will be even larger.
Geometry. All esds (except the esd in the dihedral angle between two l.s. planes) are estimated using the full covariance matrix. The cell esds are taken into account individually in the estimation of esds in distances, angles and torsion angles; correlations between esds in cell parameters are only used when they are defined by crystal symmetry. An approximate (isotropic) treatment of cell esds is used for estimating esds involving l.s. planes.
Refinement. Refinement of F^2^ against ALL reflections. The weighted R-factor wR and goodness of fit S are based on F^2^, conventional R-factors R are based on F, with F set to zero for negative F^2^. The threshold expression of F^2^ > 2sigma(F^2^) is used only for calculating R-factors(gt) etc. and is not relevant to the choice of reflections for refinement. R-factors based on F^2^ are statistically about twice as large as those based on F, and R- factors based on ALL data will be even larger.

### Fractional atomic coordinates and isotropic or equivalent isotropic displacement parameters (Å^2^)

**Table:**

	*x*	*y*	*z*	*U*_iso_*/*U*_eq_	Occ. (<1)
O	0.2331 (4)	0.50956 (7)	0.86392 (12)	0.0677 (5)	
N1	0.4420 (4)	0.25146 (8)	0.92913 (12)	0.0454 (4)	
N2	0.4631 (4)	0.39274 (9)	0.95953 (12)	0.0487 (5)	
H2A	0.5892	0.3784	1.0248	0.058*	
C1	0.4561 (6)	0.10020 (12)	0.92021 (19)	0.0675 (6)	
H1A	0.6045	0.1096	0.9939	0.101*	0.50
H1B	0.5773	0.0685	0.8621	0.101*	0.50
H1C	0.2560	0.0696	0.9407	0.101*	0.50
H1D	0.3540	0.0556	0.8706	0.101*	0.50
H1E	0.3812	0.0967	1.0024	0.101*	0.50
H1F	0.7025	0.0955	0.9237	0.101*	0.50
C2	0.3474 (5)	0.18289 (10)	0.86378 (16)	0.0482 (5)	
C3	0.1571 (5)	0.18820 (12)	0.75053 (17)	0.0548 (6)	
H3A	0.0947	0.1399	0.7064	0.066*	
C4	0.0617 (5)	0.26555 (11)	0.70415 (17)	0.0552 (6)	
H4A	−0.0665	0.2698	0.6283	0.066*	
C5	0.1561 (5)	0.33684 (12)	0.77004 (15)	0.0489 (5)	
H5A	0.0949	0.3899	0.7405	0.059*	
C6	0.3460 (5)	0.32573 (10)	0.88206 (15)	0.0421 (5)	
C7	0.4079 (5)	0.47496 (11)	0.94694 (16)	0.0460 (5)	

### Atomic displacement parameters (Å^2^)

**Table:**

	*U*^11^	*U*^22^	*U*^33^	*U*^12^	*U*^13^	*U*^23^
O	0.0921 (11)	0.0427 (8)	0.0626 (9)	0.0079 (7)	−0.0296 (8)	0.0000 (6)
N1	0.0530 (10)	0.0360 (9)	0.0464 (9)	0.0001 (8)	−0.0011 (7)	−0.0017 (7)
N2	0.0639 (11)	0.0342 (9)	0.0452 (8)	0.0019 (8)	−0.0138 (7)	−0.0021 (7)
C1	0.0770 (16)	0.0412 (12)	0.0839 (14)	0.0009 (11)	0.0040 (12)	−0.0021 (10)
C2	0.0500 (12)	0.0391 (10)	0.0562 (11)	−0.0025 (9)	0.0076 (9)	−0.0051 (9)
C3	0.0605 (13)	0.0481 (12)	0.0558 (11)	−0.0083 (10)	0.0035 (10)	−0.0146 (9)
C4	0.0585 (13)	0.0594 (13)	0.0461 (10)	−0.0065 (11)	−0.0056 (9)	−0.0078 (9)
C5	0.0549 (12)	0.0467 (11)	0.0438 (9)	0.0028 (10)	−0.0052 (9)	0.0006 (9)
C6	0.0470 (11)	0.0371 (10)	0.0419 (9)	−0.0002 (9)	0.0016 (8)	−0.0026 (8)
C7	0.0551 (12)	0.0377 (11)	0.0441 (10)	0.0024 (9)	−0.0038 (9)	0.0005 (8)

### Geometric parameters (Å, °)

**Table:**

O—C7	1.217 (2)	C1—H1E	0.9600
N1—C6	1.333 (2)	C1—H1F	0.9600
N1—C2	1.340 (2)	C2—C3	1.386 (3)
N2—C7	1.335 (2)	C3—C4	1.373 (3)
N2—C6	1.413 (2)	C3—H3A	0.9300
N2—H2A	0.8600	C4—C5	1.378 (2)
C1—C2	1.502 (3)	C4—H4A	0.9300
C1—H1A	0.9600	C5—C6	1.383 (2)
C1—H1B	0.9600	C5—H5A	0.9300
C1—H1C	0.9600	C7—C7^i^	1.532 (3)
C1—H1D	0.9600		
			
C6—N1—C2	117.86 (14)	H1C—C1—H1F	141.1
C7—N2—C6	129.90 (15)	H1D—C1—H1F	109.5
C7—N2—H2A	115.1	H1E—C1—H1F	109.5
C6—N2—H2A	115.1	N1—C2—C3	121.61 (16)
C2—C1—H1A	109.5	N1—C2—C1	116.48 (16)
C2—C1—H1B	109.5	C3—C2—C1	121.91 (16)
H1A—C1—H1B	109.5	C4—C3—C2	119.32 (17)
C2—C1—H1C	109.5	C4—C3—H3A	120.3
H1A—C1—H1C	109.5	C2—C3—H3A	120.3
H1B—C1—H1C	109.5	C3—C4—C5	119.96 (17)
C2—C1—H1D	109.5	C3—C4—H4A	120.0
H1A—C1—H1D	141.1	C5—C4—H4A	120.0
H1B—C1—H1D	56.3	C4—C5—C6	116.85 (16)
H1C—C1—H1D	56.3	C4—C5—H5A	121.6
C2—C1—H1E	109.5	C6—C5—H5A	121.6
H1A—C1—H1E	56.3	N1—C6—C5	124.40 (15)
H1B—C1—H1E	141.1	N1—C6—N2	112.23 (14)
H1C—C1—H1E	56.3	C5—C6—N2	123.37 (15)
H1D—C1—H1E	109.5	O—C7—N2	126.71 (17)
C2—C1—H1F	109.5	O—C7—C7^i^	121.3 (2)
H1A—C1—H1F	56.3	N2—C7—C7^i^	111.96 (19)
H1B—C1—H1F	56.3		

Symmetry codes: (i) −*x*+1, −*y*+1, −*z*+2.

### Hydrogen-bond geometry (Å, °)

**Table:**

*D*—H···*A*	*D*—H	H···*A*	*D*···*A*	*D*—H···*A*
N2—H2A···O^i^	0.86	2.24	2.6718 (18)	111

Symmetry codes: (i) −*x*+1, −*y*+1, −*z*+2.

## References

[bb1] Hsu, Y.-F. & Chen, J.-D. (2004). *Eur. J. Inorg. Chem.* pp. 1488–1493.

[bb2] Hu, H.-L., Yeh, C.-W. & Chen, J.-D. (2004). *Eur. J. Inorg. Chem.* pp. 4696–4701.

[bb3] Sheldrick, G. M. (2008). *Acta Cryst* A**64**, 112–122. 10.1107/S0108767307043930 18156677

[bb4] Siedel, M. C., Tuyle, G. C. V. & Weir, W. D. (1970). *J. Org. Chem.* **35**, 1662–1664.

[bb5] Siemens (1995). *XSCANS* Siemens Analytical X-ray Instruments Inc., Madison, Wisconsin, USA.

